# Interaction vs. observation: distinctive modes of social cognition in human brain and behavior? A combined fMRI and eye-tracking study

**DOI:** 10.3389/fnhum.2012.00331

**Published:** 2012-12-19

**Authors:** Kristian Tylén, Micah Allen, Bjørk K. Hunter, Andreas Roepstorff

**Affiliations:** ^1^The Interacting Minds Group, Center for Functionally Integrative Neuroscience, Aarhus UniversityAarhus, Denmark; ^2^Department for Aesthetics and Communication, Center for Semiotics, Aarhus UniversityAarhus, Denmark; ^3^Department for Culture and Society, Aarhus UniversityAarhus, Denmark

**Keywords:** social interaction, brain imaging, theory of mind, mirror neuron system, joint action, coupled dynamics

## Abstract

Human cognition has usually been approached on the level of individual minds and brains, but social interaction is a challenging case. Is it best thought of as a self-contained individual cognitive process aiming at an “understanding of the other,” or should it rather be approached as an collective, *inter*-personal process where individual cognitive components interact on a moment-to-moment basis to form coupled dynamics? In a combined fMRI and eye-tracking study we directly contrasted these models of social cognition. We found that the perception of situations affording social contingent responsiveness (e.g., someone offering or showing you an object) elicited activations in regions of the right posterior temporal sulcus and yielded greater pupil dilation corresponding to a model of coupled dynamics (joint action). In contrast, the social-cognitive perception of someone “privately” manipulating an object elicited activation in medial prefrontal cortex, the right inferior frontal gyrus and right inferior parietal lobus, regions normally associated with Theory of Mind and with the mirror neuron system. Our findings support a distinction in social cognition between *social observation* and *social interaction*, and demonstrate that simple ostensive cues may shift participants' experience, behavior, and brain activity between these modes. The identification of a distinct, interactive mode has implications for research on social cognition, both in everyday life and in clinical conditions.

## Introduction

Recent advances in evolutionary anthropology and experimental psychology suggest that one of the keys to the unique evolutionary trajectory of the human species can be found in our advanced capacities for reciprocal social interaction (Donald, 1991, 2001; Tomasello, 1999, 2008; Tomasello et al., 2005; Csibra and Gergely, 2009, 2011). This inevitably leads to fundamental questions concerning the neurocognitive foundations of such social capacities. During the last couple of decades, an increasing number of studies have addressed the human brain mechanisms responsible for our ability to make sense of social phenomena. A number of brain networks—often referred to as “the social brain”—are found to be associated with various aspects of social cognition. For instance, the medial prefrontal and temporo-parietal cortices consistently activate in tasks involving Theory of Mind/mentalizing (e.g., Castelli et al., 2000; Gallagher et al., 2000; German et al., 2004; Walter et al., 2004), while premotor areas and inferior parietal cortices seem to be involved in mental mirroring of others' motor actions (e.g., Arbib et al., 2000; Rizzolatti et al., 2001; Stamenov and Gallese, 2002; Heiser et al., 2003; Kaplan and Iacoboni, 2006; Ocampo et al., 2011). While these studies make up an intriguing body of research on the neurobiological foundations of what we might term “social observation” (where no contingent response is afforded), it is disputable to which degree the findings can be generalized to account for processes underlying social interaction. We argue that the distinction between 3rd person social observation and 2nd person social interaction is an important conceptual and empirical distinction that has been somewhat neglected in the neurocognitive field (Roepstorff, 2001; Tylén and Allen, 2009; Schilbach, 2010; Hasson et al., 2012).

Two prevalent conceptual frameworks have oriented the majority of studies in social neurocognition, Theory of Mind/mentalizing (hence ToM) and Simulation Theory (which is often closely associated with the Mirror-System hypothesis—hence MNS). In both cases, the overall goal is to unravel and map the neurobiological mechanisms responsible for the ability to attribute, understand, and empathize mental states of others. Although we recognize that the underlying assumptions and proposed mechanisms of ToM and MNS are indeed very different, they take the same point of departure: the individual mind. ToM and MNS models are thus mainly preoccupied with the way individuals make sense of each other from an observational point of view (Gallagher and Hutto, 2008). The fundamental processes of social cognition are described in terms of mental inference (ToM) or embodied simulation (MNS) facilitating a “self-contained understanding” of other persons' actions. This “understanding” in turn supposedly makes it possible to choose appropriate responses, and for instance engage in *inter*actions (Frith and Frith, 2001, 2006a; Schulte-Ruther et al., 2007). In other words, individual observational processes are—more or less explicitly—given primacy as constituting the core of social cognition, while other social cognitive phenomena (e.g., social interaction) are derived from or emergent upon these fundamental processes. Hence in these frameworks, mechanisms in social interaction are extrapolated from studies of social observation and thus explained on the level of individual minds and brains. An interaction thus involves two or more individuals that recursively observe, represent and react to each other's actions based on their individual internal representational models. This has important implications for the theoretical and experimental foci of the two paradigms. Here, we will make the case that social observation and social interaction are in fact very different phenomena. While an individualistic and observational stance to social cognition may be appropriate for the study of a range of phenomena including the detection of deception, pretense, emotional expressions, etc., it is much less clear to which extent it can tackle questions related to the inherently collective and reciprocal dynamics of *social interaction*.

A growing literature within philosophy of mind and cognitive science is advancing the view that in order to adequately account for cognitive processes involved in social interaction, we need to widen the perspective beyond individual minds and brains. These approaches are largely informed by recent discussions under the headline of “extended,” “enacted,” and “distributed” cognition often relying on insights from complex systems theory. The main argument is that when two persons engage in joint activities their bodies, actions, and individual cognitive processes become coupled in dynamic ways. Hence rather than working in parallel as self-enclosed autonomous entities, persons involved in direct interaction get intermingled in complementary ways that enable emergent synergies (De Jaegher et al., 2010; Hasson et al., 2012). In this understanding, a sequence of joint action is better conceived of as a whole (singular, continuous) time series, rather than a synchronization of two independent processes (Black et al., 2007; Konvalinka et al., 2010; Riley et al., 2011). As an example, consider a dialogue. In conversation, interlocutors take turns in a complementary way making up the overall object of the dialog. One interlocutor's speech turn—for example, a question—is only completed by the responding speech turn of the other (cf. the concept of “adjacency pairs,” Goodwin and Heritage, 1990). If we isolate an individual component, say all the speech turns of one interlocutor, we are left with a partial object that does not make any sense on its own. In other words, the dialog as a phenomenon cannot be reduced to any of the partial individual components, but can only be appropriately assessed at the collective, inter-personal level (Kello et al., 2010). We argue that turn-taking-like responsiveness is a fundamental characteristic of social interaction across a broad range of contexts from diaper-changing to tango-dancing. As a distinct phenomenon, it should not be confused with automatic mirroring or simulation. Where mirroring is assumed to be an internal representational event, turn-taking responsiveness is rather characterized by its complementary contribution to the intersubjective scene. The ostensive act of one person (e.g., a greeting nod or an eyebrow flash) afford for the complementary response from the recipient (e.g., an “answering” nodding gesture). An offering hand gesture affords a receptive one (Newman-Norlund et al., 2007; Ferri et al., 2011; Sartori et al., 2012).

Which predictions follow from the conceptual approach to social interaction sketched above? If key dynamics of social interaction can only be found at a collective, level, how can we then study its neurocognitive underpinnings? One suggestion is that simultaneous recording from multiple agents is necessary to make claims about the dynamics of mutually coupled cognitive systems. While this may be a useful approach (see Konvalinka and Roepstorff, 2012) we here argue that recognizing the coordinative nature of social interaction allows specific predictions, even on the level of individual brains recorded in isolation. If the brain in joint action becomes a component-node in a larger interactive array, we can reframe the basic question as: What does it take for a brain to successfully engage in reciprocal coupling processes with other responsive components?

For a component to successfully work in tight concert with other external components it has to continuously integrate, adapt and respond to incoming stimuli at a multiplicity of temporal levels and modalities (Konvalinka et al., 2010). This suggests that rapid adaptation and coordination are crucial factors in real-time interaction. These properties are fundamentally different from those involved in “social observation.” Where an observational understanding of a social phenomenon may be internally realized in terms of simulation or inference, a socially interactive practice calls for moment-to-moment reciprocity with one or more co-operative partners in the “external” social environment.

These fundamental differences between social observation and social interaction predict the involvement of distinct anatomical structures in the two processes. Regions of the temporal lopes (in particular STS, pSTS) have been consistently associated with the fine-grained continuous temporal integration of dynamic stimuli (Hasson et al., 2008; Stephens et al., 2010; Lerner et al., 2011). These structures, particularly in the right hemisphere, have indeed been found in a number of recent studies addressing the neurocognitive underpinnings of joint action and joint attention. In a fMRI study conducted by Newman-Norlund et al. (2008), activity was enhanced in right pSTS when participants performed a joint task with another person in the control room affording complementary (non-isomorphic) actions. In a study by Redcay et al. (2010), participants underwent fMRI scanning while solving a cooperative joint attention task with another person through a bidirectional video link. Again the main findings related to right pSTS/TPJ. Likewise, a fMRI study applying a dual player virtual communication game (Noordzij et al., 2009) also found the right pSTS to be modulated by social interaction in contrast to solo conditions, and finally a study by Iacoboni et al. (2001) found that the right pSTS was more active when participants imitated displayed hand movement than when they produced them from memory. We notice that the rpSTS has both been argued to belong to the ToM network (Frith and Frith, 2006b) and to the MNS (Van Overwalle and Baetens, 2009). However, while pSTS may co-activate with both of these networks in task specific ways, no consistent pattern has so far been established, and no stable connectivity has been established between the pSTS and regions associated with ToM and MNS (Ethofer et al., 2011). We thus argue that pSTS is not a constitutive part of the ToM or the MNS network.

The findings cited above indicate the right pSTS as an area particularly sensitive to the continuous fine-grained temporal navigation and integration of stimuli necessary for immediate contingent responsiveness in social interaction. Thereby, it seems a good anatomical candidate for our hypothesized distinctive mode of social engagement. We thus predict that social interaction will recruit the pSTS, while social observation primarily will rely on networks related to ToM and MNS. How can we test such hypotheses?

This requires an experimental paradigm that directly compares interactive and observational social cognition. Here, we report an fMRI experiment that contrasts video stimuli, which either evoked an observational or interactive responsive attitude in the participant toward an actor performing simple object-related gestures. This contrast was established by modulating the ostensive character of the performed action. In the interactive conditions, the actor made interaction initiation cues (eye contact, eyebrow flashes and nods) before performing a *placing-object-for* or *showing-object-to* action (Clark, 2005). In contrast, in the non-interactive “private” condition the same actions were performed without ostensive cues. Moreover, the directionality of the action was modulated so that in some conditions the actor would face the participant while in others she/he was presented from a slightly averted perspective as if facing someone outside the perspective of the camera.

The theoretical analysis above generated specific anatomical hypotheses relating to three clusters of brain areas associated with ToM (in particular MPFC and TPJ), the MNS (pre-Motor, IPL), and Joint Action/Joint Attention (pSTS). We thus restricted the study to target these particular areas using a ROI approach (see section “Materials and Methods” for details). We predicted that ostensive object-gestures would engage contingent responsiveness in the participants, and that this would elicit differential activation in pSTS. In contrast, observing “private” object manipulations would evoke an observational attitude in the participant and hence elicit activations in ToM and MNS regions. Beside, we hypothesized that activity in these areas would be modulated by the directionality of action, as either participant-directed or other-directed.

Since the pSTS has also been associated with perspective taking, eye-gaze and saccading behaviors (Allison et al., 2000), we included simultaneous in-scanner-eye-tracking to control for effects caused by participants' simple eye gaze-behaviors. Furthermore, we used pupillometrics (pupil size measurements) to assess pupil dilation and constrictions in response to the experimental conditions (Kampe et al., 2003; Granholm and Steinhauer, 2004). We predicted that interactively engaging stimuli would be more emotionally arousing resulting in greater pupil dilation than stimuli affording a more observational attitude in the participant.

## Materials and methods

### Subjects

Twenty-two healthy, right-handed adult volunteers (12 females/10 males, mean age 25 ± 4.8 STD) who had all given their written consent in correspondence with the requirements of the local ethical committee participated in the experiment. The participants were mainly recruited among students at Aarhus University, and were naïve with respect to the purpose of the study.

### Stimuli and experimental design

**Figure 1:**
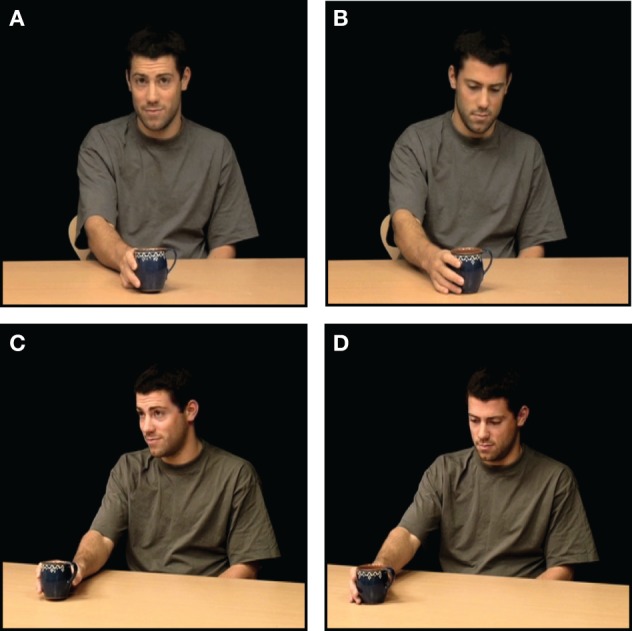
**Example of stimuli.** In 5 s video clips, an actor performed simple object gestures (“*placing an object for”* or “*showing an object to*” someone) in four conditions: **(A)** ostensive and direct, **(B)** non-ostensive and direct, **(C)** ostensive and averted, **(D)** non-ostensive and averted. Besides, all four conditions were replicated without the object gesture.

Stimuli consisted of 32 video clips of 5 s duration, showing an actor sitting at a table in front of an object (see Figure 1). The videos differed on three variables: (1) actor gender (m/f), (2) object (cup or fruit) and—for the action condition—(3) action type (*placing-object-for* or *showing-object-to*) (cf. Clark, 2005). The experiment was divided into two sessions of 64 trials (i.e., all videos were shown four times).

We used two-by-two-by-two factorial design (making up in all eight conditions) with the main factors Ostention (ostensive/non-ostensive), Direction (direct/diverted perspective), and Action (action/no action). In ostensive conditions, the actor would look up and make an interaction-initiating cue by establishing eye contact (either to the participant or to an inferred other outside the scope of the camera) and making an eyebrow lift and a nod before performing one of the two object directed gestures. In non-ostensive conditions the action was performed “privately” without any addressing cues or eye contact. In direct conditions, the ostensive cues and gestures were performed directly to the participant (i.e., the camera), while in the diverted condition the actor was oriented at approx. 20° of the camera in the direction of an inferred other (see Figure 1). In the no action conditions, the four conditions above were replicated, but without the object gesture.

The stimulus videos were presented in blocks of two clips from the same condition, and the order of blocks was randomized between participants. After the two clips participants were asked one of two yes/no questions: “was it the same person” or “was it the same object?” This probed whether the same or different actors or objects had appeared in the two movie clips. The questions were randomized so that the participant could not anticipate if she/he would be asked about the actor or the object. In order to solve the task, the participant thus had to pay close attention to both actors and objects during the stimulus presentation. Participants would respond by pressing one of two buttons with their right hand index and middle finger. The left/right position of the affirmative response was randomized across trials.

### Scanning parameters

We used a 3T General Electrics MR system (Waukesha, WI, USA) with an eight channel head coil to acquire the T^2^ -weighted gradient, echo-planar images (EPI) with Blood Oxygenation Level-Dependent (BOLD) contrast using the following parameters: echo time (TE): 30 ms, repetition time (TR): 3000 ms, and a flip angle of 90°. Whole-brain images were obtained over 39 sequential, interleaved 3.5 mm axial slices with a 128 × 128 pixel resolution matrix and a field of view of 240 × 240 mm.

### Eye-tracking parameters

Participants' eye movements and pupil size were recorded simultaneously with the MR acquisition using a SMI/Avotec IViewX eye-tracking system in a Silent Vision 7021 MR-insert binocular visual system. Data were recorded from the right eye with a sample frequency of 50 Hz. Prior to each of the two scanning sessions, the eye-tracker was calibrated using the IViewX nine-point automated calibration procedure, which was repeated until the calibration was satisfactory. The eye-tracker was linked and synchronized with the MR stimulus computer and continuously recorded time stamps for the initiation of stimulus videos.

### Additional behavioral testing

After the fMRI scanning, participants went through an extensive debriefing where they evaluated their experience on various parameters. Moreover, participants watched the stimulus videos again on a computer screen and rated how “socially engaging” they found them on a 5 point scale where 1 = not engaging, and 5 = very socially engaging.

## Analysis

### Behavioral analyses

Task performance (response accuracy) from the in-scanner task was summarized and averaged for each participant and tested against chance performance using paired *t*-tests. Likewise, the post-scanning ratings of the socially engaging nature of the stimuli were summarized and averaged for each participant and each condition, and condition-related differences were tested using a within-subject, repeated measures, three-way analysis of variance (Howell, 2002), with the factors *Ostension* (+/−), *Direction* (direct/diverted), and *Action* (+/−). The analysis was thresholded at *p* < 0.05. Due to technical problems, we failed to obtain rating data from two participants, so only data from the remaining twenty participants entered this analysis. All statistical tests were performed in MATLAB 2011b.

### Eye-tracking analysis

Since the experiment was mainly optimized for fMRI acquisition, only full eye-tracking data sets from eleven participants entered the analysis. The remaining data were lost or corrupted due to technical problems and calibration difficulties. Task related eye-tracking data (*x*/*y* coordinates and *x*/*y* pupil diameter in pixels at a 50 Hz sampling for each 5 s stimulus video) were preprocessed by removing eye blinks and outliers (deviating in distance with more that 3 × SD of the mean). The data were high-pass filtered at a 100 s cut off to counter calibration drift. Saccade velocity was then calculated for each participant based on point-to-point Euclidean distance (Salvucci and Goldberg, 2000). Similarly, pupil diameter was calculated as an average of the pupil *x* and *y* diameter direction (although these were strongly correlated we used this procedure to get a more stable index of pupil size). Velocity and pupil size data were averaged for each stimulus trial before entering further analysis.

The preprocessed data were used in two ways: first, to test for condition related differences in participants' gaze behaviors (hence the “stand-alone eye-tracking analysis”). For this purpose eye-movement velocity and pupil size data from each condition entered a within-subject, repeated measures, three-way analysis of variance (Howell, 2002), with the factors *Ostension* (+/−), *Direction* (direct/diverted), and *Action* (+/−). The analysis was thresholded at *p* < 0.05. Second, for each participant, velocity data were averaged for each stimulus event to be included as a first-level parametric modulation in the fMRI analysis (hence “the combined eye-tracking/fMRI analysis”).

### fMRI analysis

All fMRI data analysis was conducted using SPM8 (Statistical Parametric Mapping, Wellcome Department of Imaging Neuroscience, London) implemented in MATLAB 2011b (Mathworks Inc. Sherborn, MA) using default settings unless otherwise specified. Images were spatially realigned, normalized to the MNI template and smoothed with an isotropic 8 mm FWHM Gaussian kernel.

Statistical analysis was conducted following a two-level general linear model approach (Penny and Holmes, 2007). On the first-level, task related BOLD responses were modeled for each subject by convolving condition onsets and durations with the standard hemodynamic response function and contrasting factorial main and interaction effects. Two independent first-level analyses were carried out. The first, which was carried out for all participants, included a regressor (parametric modulation) for each of the variables of the stimulus videos (gender, object and action type) as well as the six standard SPM8 motion parameters. The second first-level analysis was only carried out on data from the 11 participants from who we recorded a full eye-tracking data set. In addition to the stimulus and motion regressors used in the analysis above, this analysis included a parametric modulation regressing out relative differences in participants' eye-movements (saccade activity). For both first-level analyses, images were high-pass filtered at a 128 s cut off.

#### Second level RFX analyses

Two group RFX analyses were conducted—one for each of the first-level analyses—using a Three-Way repeated measures whole brain ANOVA (corrected for non-sphericity) in SPM8. The directionality of effects was explored using one-sample *t*-tests. In both cases, individual subject effects were modeled using the covariate function to adjust the statistics and degrees of freedom during inference. We did not assume independence or equal variance (Christensen and Wallentin, 2011). For both analyses, the significance threshold was set to *p* < 0.05, FWE corrected for multiple comparisons. Functional images were overlaid with the standard SPM8 single subject high resolution T1 image.

To constrain the analyses to specific, predefined anatomical sites (see section “Introduction” above) for which we had hypotheses, we used a *region of interest* (ROI) approach. The analyses were carried out as small volume corrections by masking particular brain structures consistently found in neurocognitive studies on social cognition. Masks were generated in the Wake Forest University PickAtlas extension for SPM (Tzourio-Mazoyer et al., 2002; Maldjian et al., 2003, 2004) as 10 mm spheres centered in target peak voxels. These were reported as main findings in recent studies on closely related topics all using stimuli very compatible to ours (dynamic video stimuli displaying an actor performing different types of actions). We recognize that a number of other areas have previously been reported as associated to ToM and MNS, but we chose to restrict our self to a few canonical areas of the right hemisphere which are among the most consistently reported in the literature and that have been associated with tasks resembling ours. The following masks were employed: to test for neural activity related to ToM/mentalizing we masked regions in mPFC [MNI (13, 37, 2)] and rTPJ [MNI (49, −63, 29)] based on coordinates from Wurm et al. (2011). To test for neural activity related to the mirror neuron system we masked the right IFG [MNI (52, 32, 24)] and IPL [MNI (46, −48, 44)] based on coordinates from Ocampo et al. (2011). Finally, to test for neural activity related to joint action/attention, we masked right pSTS [MNI (48, −40 6,)], based on coordinates from Redcay et al. (2010).

## Results

### Behavioral results

Participants were generally able to solve the in-scanner recollection task (“same actor/same object?”), and scored an average response accuracy of 72% (*SD* = 5.33). One participant did not perform significantly above chance due to a high number of missed responses. However, since the participant did not self-report concentration/sleepiness problems etc., and the exclusion of the data did not affect the analysis substantially, the participant was not excluded from the fMRI analysis.

**Figure 2:**
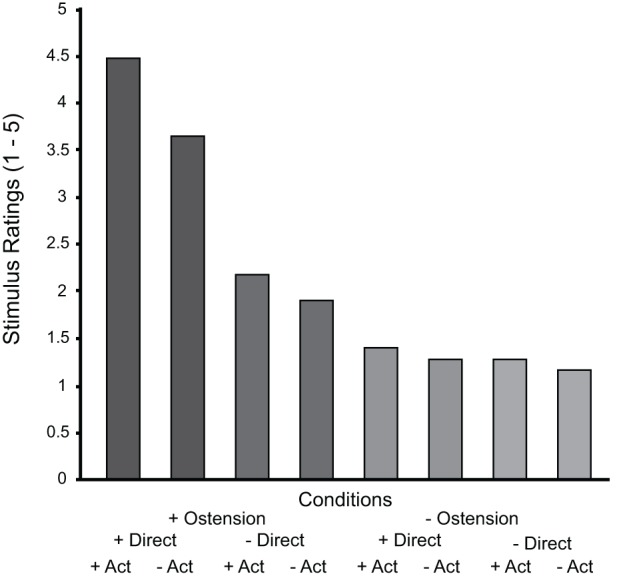
**Results from the post-scan stimulus ratings sorted by condition.** Notice that the graph summarizes the “within-subject” results across all subjects and therefore we have not included error bars (between subject variance would not reflect the actual analysis).

The post-scanning rating of the socially engaging nature of the stimulus videos showed a number of significant between-condition differences and interactions. The main effect of ostension yielded an *F* ratio of *F*_(1, 19)_ = 203, *p* < 0.000, indicating that the ostensive behavior of the actor made the scenes overall more socially engaging (*M* = 3.05, *SD* = 0.77) than non-ostensive scenes (*M* = 1.28, *SD* = 0.35). The main effect of direction was also significant, *F*_(1, 19)_ = 10.5, *p* < 0.01, likewise indicating that direct perspective was found more socially engaging (*M* = 2.69, *SD* = 0.44) than diverted perspective (*M* = 1.63, *SD* = 0.68). Finally, the main effect of action was also found significant: *F*_(1, 19)_ = 23.4, *p* < 0.000, indicating that the more dynamic scenes including object manipulations (placing for/showing) were more socially engaging (*M* = 2.32, *SD* = 0.58), than non-dynamic scenes (*M* = 1.99, *SD* = 0.53). Besides, all interactions were significant. Ostension interacted thus significantly with direction: *F*_(1, 19)_ = 54.2, *p* < 0.000, indicating that scenes were found more socially engaging when ostensive cues were performed directly to the participant. Ostension interacted significantly with action: *F*_(1, 19)_ = 56.6, *p* < 0.000, suggesting that the scenes were found more socially engaging when ostension and action accompanied each other (to form communicative gestures). Direction and action also interacted significantly, although to a somewhat lesser extent: *F*_(1, 19)_ = 7.2, *p* < 0.05, and, finally, the factors showed a significant three-way interaction: *F*_(1, 19)_ = 10.1, *p* < 0.005 (see Figure 2).

### Stand-alone eye-tracking results

The analysis of participants' eye-movements (saccade velocity) related to the conditions showed some significant effects. The main effect of direction yielded an *F* ratio of *F*_(1, 10)_ = 8, *p* < 0.05, suggesting that participants generally displayed more saccading behaviors in the diverted conditions (*M* = 0.31, *SD* = 0.09) than in the direct (*M* = 0.29, *SD* = 0.08). The main effect of action was also found significant: *F*_(1, 10)_ = 19.2, *p* < 0.005, indicating that participants made more saccades in the action (*M* = 0.32, *SD* = 0.09) than the no-action conditions (*M* = 0.29, *SD* = 0.08). The main effect of ostension and all interaction effects were non-significant.

**Figure 3:**
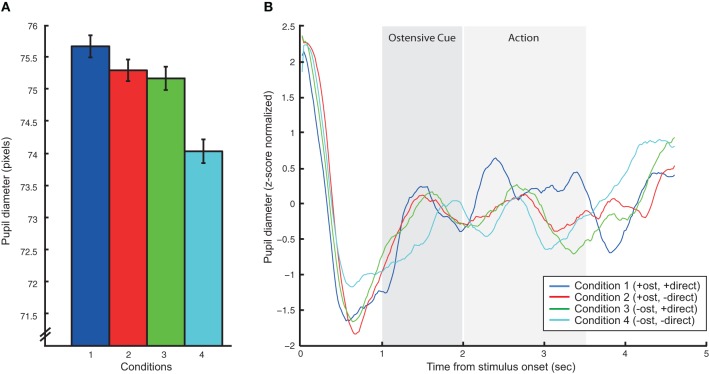
**Results of the pupillometric analysis. (A)** Summarizes mean pupil dilations in pixels for each of the four action conditions (we here omit the no-action conditions to simplify the graph since that main effect of action was not significant). Error bars express standard error of the mean. **(B)** Shows averaged, baselined pupil dilation/constriction patterns for each of the four action conditions over the time course of the 5 s stimulus videos. Vertical gray bars putatively indicate the onset and duration of ostensive cues and action.

The analysis of pupil diameter changes also showed significant effects. The main effect of ostension yielded an *F* ratio of *F*_(1, 10)_ = 5.2, *p* < 0.05, indicating pupil dilation (measured in pixels) in response to ostensive cues (*M* = 78, *SD* = 10.7) relative to non-ostensive scenes (*M* = 77.7, *SD* = 10.6). Likewise, the main effect of direction was found significant: *F*_(1, 10)_ = 18.4, *p* < 0.005, suggesting dilation in response to direct perspective (*M* = 78.4, *SD* = 10.8) relative to diverted perspective (*M* = 77.3, *SD* = 10.5). The main effect of action had no effect on pupil size and all interaction effects were non-significant (see Figure 3).

### fMRI results

**Figure 4:**
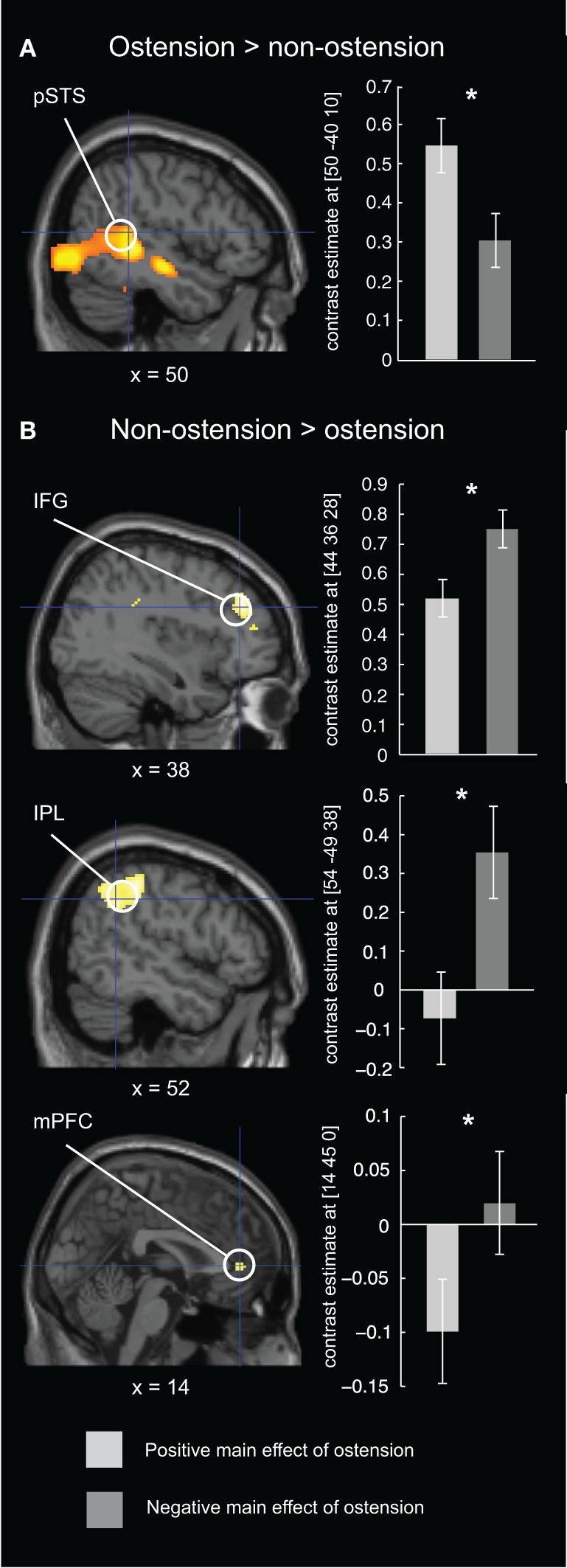
**Results of the fMRI ROI analysis for the ostensive condition.** Left column: brain maps depicting differential BOLD responses evoked by ostension (+/−) in relevant ROIs. Right column: bar plot of peak voxel contrast estimates for the positive and negative main effect of ostension in each of the ROIs. Error bars express 90% confidence intervals. **(A)** in the positive main effect of ostension, higher BOLD responses were found in rpSTS, an area related to fine temporal integration and adaptation (e.g., in the context of joint action). **(B)** in the negative main effect of ostension, higher BOLD responses were found in rIFG and rIPL, areas often associated with the mirror neuron system, and in the mPFC, often associated with theory of mind/mentalizing. ^*^p < 0.05 (FWE-corrected).

As predicted, the positive main effect of ostension significantly modulated activity in regions associated with Joint Action/Attention, i.e., the ROI in right pSTS [peak voxel: MNI (48, −38, 0)]. However, no above threshold activations were found in ROIs associated with ToM and MNS (i.e., mPFC, rTPJ, rIFG, and rIPL) (see Figure 4A and Table 1). In contrast, the negative main effect of ostension was found significant in a number of ROIs related both to ToM and MNS: mPFC [peak voxel: MNI (6 42 0)], rIPL [MNI (54, −48, 38)], and rIFG [MNI (44, 36, 28)] (see Figure 4B and Table 1). No significant effects were found in the rTPJ and pSTS for this contrast.

**Table 1:** **fMRI results (ROI analysis)**.

**Putative anatomical regions**	***Z*-scores**	**Coordinates**		
		***x***	***y***	***z***
**OSTENSION > NON-OSTENSION**				
pSTS (right)	4.61	48	−38	0
**NON-OSTENSION > OSTENSION**				
IPL (right)	3.86	54	−48	38
IFG/MFG (right)	3.23	44	36	28
mPFC	2.99	6	42	0
**ACTION > NON-ACTION**				
pSTS (right)	7.78	52	−48	2
IPL (right)	4.19	42	−44	52
IFG/MFG (right)	5.16	58	30	30

Main effects of experimental conditions (t-statistics) thresholded at p < 0.05 (FWE corrected for multiple comparisons).

The main effect of direction (both positive and negative) did not modulate activity in any of the predefined ROIs. However, explorative whole-brain analysis revealed activity in early visual areas (V1) possibly related to participants' increased eye movements in this condition (see section “Stand-Alone Eye-Tracking Results” above). These results will thus not be considered any further.

The positive main effect of action elicited significant activity in a number of ROIs relating to the MNS and Joint Action: right pSTS [peak voxel: MNI (52, −48, 2)], rIPL [MNI (42, −44, 52)], and rIFG [MNI (58, 30, 30)] (see Table 1). However, ROIs associated with ToM (mPFC and rTJP) did not give significant results. The negative main effect of action did not show any effects. Likewise, none of the interaction effects showed significant results.

#### Eye-movement corrected fMRI results

When factoring in parametric modulations expressing participants' relative eye-movements, activation patterns largely resemble the results from the analysis above. This indicates that the results reported in Table 1 are not confounded by condition-related differences in participants' eye-movement patterns. However, the overall statistical strength is considerably weaker, possibly due to the reduced number of participants entering this analysis (full data sets could only be obtained from 11 participants). The positive main effect of ostension was significant in right pSTS [peak voxel: MNI (46, −40, 6)], but not in any of the remaining ROIs. The negative main effect of ostension did not give any above threshold activity. However, an exploratory lowering of significance thresholds revealed strong trends in mPFC [MNI (14, 28, −2), *p* = 0.005, uncorrected] and rIPL [MNI (54, −48, 48), *p* = 0.01, uncorrected] (see Table 2). No results were found in pSTS, rTPJ and rIFG.

**Table 2:** **Eye-movement corrected fMRI results (11 participants)**.

**Putative anatomical regions**		***Z*-scores**	**Coordinates**		
			***x***	***y***	***z***
**OSTENSION > NON-OSTENSION**					
rpSTS	*p* < 0.000, corrected	4.36	46	−40	6
**NON-OSTENSION > OSTENSION**					
rIPL	*p* = 0.01, uncorrected	2.34	54	−48	48
mPFC	*p* = 0.005, uncorrected	2.57	14	28	−2
**ACTION > NON-ACTION**					
rpSTS	*p* < 0.000, corrected	4.37	46	−42	6
rIPL	*p* = 0.01 corrected	3.52	38	−44	48
rTPJ	*p* = 0.01 corrected	3.53	50	−64	20

Main effects of experimental conditions (t-statistics).

The main effect of action was found to significantly modulate activity in right pSTS [MNI (46, −42, 6)], in rIPL [MNI (38, −44, 48)], and the rTPJ [MNI (50, −64, 20)] (see Table 2). No effects were found in the remaining ROIs and for the negative main effect of action. Likewise, none of the interaction effects reached above threshold significance.

## Discussion

Which brain structures facilitate contingent complementary coordination between interacting individuals? This study attempts to make an experimental contribution to current disputes concerning the foundations of social interaction. Based on recent directions in philosophy of mind and complex systems approaches we argue that social interaction may be conceptualized as a collective, interpersonal phenomenon constituted by multi-modal intersubjective coordination processes. This approach departs from ToM and MNS based frameworks, where interaction is founded on or extrapolated from individual processes of social observation, and it allows for specific predictions regarding individual brain activity during social interaction.

Participants were presented with dynamic situations that afforded different styles of social perception. In some situations, an actor “privately” manipulated objects in a non-ostensive context, while in others object gestures were accompanied with interaction-initiating, ostensive cues. Our results demonstrate that the ostensive contextualization of action radically altered the perceptual attitude of participants. While the non-ostensive scenes called for an observational attitude concerned with “understanding” the actions and intentions of the actor, the ostensive act of *placing an object for* or *showing an object to* someone strongly affords complementary completion by the recipient. The non-ostensive and ostensive scenes thus engage the participants in fundamentally different ways as “observational bystanders” or as “potential interactive recipients.” While the first type of situation (*social observation*) can be fully described on the level of individual cognition (mental inference of simulation), the second (*social interaction*) is more appropriately approached as a continuous adaptive coupling between minds (Tylén and Allen, 2009; Hasson et al., 2012). We thus predicted quite different behavioral and neurocognitive results for the two conditions.

Participants' ratings of the socially engaging character of stimulus scenes confirm such predictions. Overall, the scores suggest that although the video stimuli are inherently unresponsive (compared to “live” interaction), they successfully evoked feelings of social contingency in the participants. By far the strongest result is obtained for the positive main effect of ostension, followed by action. Curiously, and contrary to our expectations, the effect of direction is substantially weaker, indicating that the recipient design (“facing you” vs. “facing someone else”) is less important for the participants' experience of social engagement with the displayed actor. However, there are strong interaction effects indicating that direct perspective matters for the ostensive conditions while the effect is substantially weaker for the non-ostensive conditions (see Figure 2).

Analogous results are found for the fMRI brain imaging data. Among the pre-defined regions of interest, the rpSTS was most strongly activated by scenes affording social responsiveness. In these scenes, an actor looked up and made interaction-initiating ostensive cues (eye contact, eyebrow flashes and nods). The rpSTS area has been repeatedly associated with eye-gaze (Allison et al., 2000; Pelphrey et al., 2004). In a related study, Redcay et al. (2010) suggested that uncontrolled condition related differences in participants' eye-movement patterns could potentially confound their findings. However, we employed in-scanner eye-tracking to test for eye-movement related effects. Analyses of saccade velocities did not show significant differences for ostensive/non-ostensive conditions. Beside, when participants' eye-movements were factored into the fMRI analysis, results related to rpSTS were not influenced. Our findings relating interactive conditions to activity in the pSTS can therefore not be explained simply by differences in participants' eye movements.

Other studies have indicated that rpSTS may be particularly sensitive to gaze direction, such as the distinction between direct and averted gaze (Kuzmanovic et al., 2009; Ethofer et al., 2011). In our study, we modulated the body orientation of the actor, so that she/he was either facing the participant or presented from a slightly averted perspective. Contrary to our expectations, we did not find any modulations of the rpSTS related to this contrast (we had expected direction to interact with ostension). In fact, similar to the behavioral stimulus ratings, the body orientation of the actor showed relatively weak effects. In sum, the rpSTS effects found in this study cannot be reduced to stimulus-induced differences in participants' eye-movement patterns or effects related to the actors' body and gaze directions. Instead, they suggest that rpSTS activity may be related to a socially interactive contextualization of the scene as a whole whether or not the participant was addressed as the intended recipient of the act.

Interestingly, pupillometric analyses showed a strong main effect of direction. When the actor was oriented toward the experimental participant, we recorded stronger pupil dilations relative to diverted orientations. Similar (although slightly weaker) effects were found for ostension. Pupil dilation has been reported as a reliable marker of low-level emotional arousal related to the sympathetic nervous system (Laeng et al., 2012) and has likewise been shown to provide a sensitive index of subtle and complex cognitive and affective processes (Partala and Surakka, 2003; Granholm and Steinhauer, 2004). The pupillometric findings in this study are thus taken in support of our predictions: actors' ostensive cues and direct body orientation induce participants with increased levels of attentional alertness due to affordances for complementary responsive action. It should be noted that although the activation of rpSTS does not follow the same pattern as the pupil dilations (rpSTS seems insensitive to direction), we cannot fully exclude the possibility that arousal rather than complementary interactive dynamics drives some of the brain activation patterns in this study.

Together, the findings can inform discussion between different models of social cognition. While many “observational” social cognition tasks rely solely on participants to internally represent other agents' behaviors, intentions and beliefs, social interaction is more appropriately depicted as a continuous contingent coupling between two or more individuals (Hasson et al., 2012). Right pSTS has been reported in a number of studies contrasting situations where participants solve tasks relying on continuous coordination with external social stimuli with situations where they solve tasks purely based on internal reasoning processes (Iacoboni et al., 2001; Newman-Norlund et al., 2008; Noordzij et al., 2009; Wyk et al., 2009; Redcay et al., 2010; Carter et al., 2011). A subset of these studies (Newman-Norlund et al., 2008; Noordzij et al., 2009; Redcay et al., 2010) even facilitated live contingent interaction between experimental participants lying in the scanner and cooperative partners in the control room in cooperative tasks. It should be noted that—based solely on our data—we cannot exclude the possibility that the rpSTS effect found in our study reflects a mentalizing strategy. Following the work of e.g., Gergely and colleagues (Csibra and Gergely, 2009; Southgate et al., 2009), ostensive cues can act to direct and enhance attention to a subsequent behavior and thereby facilitate “understanding of the social goal” of the agent. However, considering the growing literature associating the rpSTS with contingent social interaction, we favor the interpretation that the effect relates to the socially engaging affordances of the ostensive stimulus scenes evoking a strong inclination to respond in complementary ways (Sartori et al., 2012).

Interestingly, when participants were confronted with non-ostensive scenes featuring an actor “privately” manipulating objects, we found increased activation of areas normally associated with ToM (mPFC) and MNS (rIPL and rIFG). We notice that although the frontal component of our MNS mask was centered in the IFG (see section “Materials and Methods” above), the activation peak found in our study is slightly more anterior and thus rather resembles findings from Weissman et al. (2008) relating social observation to the DLPFC. In contrast to the rIPL activation, we will thus not make any strong claims about this frontal component in relation to the MNS. However, our findings suggest that the effects found in ToM and MNS related areas could be explained by reference to the quite different affordances of the control stimuli. The non-ostensive character of these scenes frames the participant as an observing bystander *making sense* of the scenes rather than *responding* to them. This form of “social observation” does not to the same extent depend on fine temporal coupling and coordination with the external social environment. Rather, it can be characterized as a decoupled process relying on inferential reasoning (mentalizing) and mental action simulation.

It has been argued that the MNS is indeed sensitive to socially complementary action affordances (Newman-Norlund et al., 2007). While an interesting TMS study could be interpreted in favor of this account (Newman-Norlund et al., 2010), other evidence is more mixed. We thus notice that in a study from the same lab, the strongest effect of complementary actions was seemingly found in the rpSTS (Newman-Norlund et al., 2008). Furthermore, other researchers have not been able to replicate the MNS findings for complementary actions (Kokal et al., 2009; Ocampo et al., 2011).

The differential activation and deactivation patterns found for interaction vs. observation conditions seem to resonate with findings on intrinsic variability of macroscopic networks associated with attention and social-cognitive action control. Indeed, evidence suggests that the neural apparatus supporting social observation (in particular mPFC and IPL) are directly inhibited by tasks requiring high cognitive demand and focused attention (Raichle et al., 2001; McKiernan et al., 2003; Spreng et al., 2009; Allen and Williams, 2011). Similarly, the continuous tracking and contingent responding required of social interaction may necessitate going “online” to the extent of actually de-activating networks associated with ToM and self-related cognition (Fox et al., 2005; Schilbach et al., 2008; Andrews-Hanna et al., 2010).

We also found a number of our regions of interest to be modulated by the positive main effect of action. In particular, significant activation was found in rIFG, rIPL and the rpSTS, while no effect was found in rTPJ and mPFC. While the activation of MNS related regions (rIFG and rIPL) is possibly related to participants' mirroring of displayed actions, we speculate that the ostensive properties of the object gestures themselves observed in the behavioral study (stimulus ratings) might account for the rpSTS component finding.

## Conclusion

The current study contrasted brain activity elicited by short videos, which evoked in the participants *observational* social cognition and *interactive* social cognition. The difference was triggered by the presence of ostensive cues, which open a channel of communication and interaction. Observational social cognition differentially evoked activity in regions hitherto associated with Theory of Mind (mPFC) and the Mirror Neuron Systems (IPL, IFG). Interactive social cognition differentially evoked activity in right posterior STS, a region known to be involved in continuous fine-grained temporal navigation and integration of stimuli. Brain imaging findings are supported by behavioral tests showing that participants found interactive conditions more socially engaging and pupillometric analyses indicating higher levels of arousal (pupil dilation) for interactive than observational conditions.

Our findings demonstrate that very simple cues may shift both the experience of participants and the brain activity associated with social cognition between an *observational mode* and an *interactive mode*. The identification of a distinct interactive mode of social cognition opens a new avenue for research on social cognition, both under normal conditions and in clinical disorders, such as autism and schizophrenia, characterized by disturbances in social cognition.

### Conflict of interest statement

The authors declare that the research was conducted in the absence of any commercial or financial relationships that could be construed as a potential conflict of interest.
